# Intracortical Circuits in Thalamorecipient Layers of Auditory Cortex Refine after Visual Deprivation

**DOI:** 10.1523/ENEURO.0092-17.2017

**Published:** 2017-04-06

**Authors:** Xiangying Meng, Joseph P. Y. Kao, Hey-Kyoung Lee, Patrick O. Kanold

**Affiliations:** 1 Department of Biology, University of Maryland, College Park, MD 20742; 2 Center for Biomedical Engineering and Technology, and Department of Physiology, University of Maryland School of Medicine, Baltimore, MD 21201; 3 Department of Neuroscience, Mind/Brain Institute, Johns Hopkins University, Baltimore, MD 21218

**Keywords:** Auditory cortex, intracortical, dark exposure, visual deprivation, crossmodal, mouse, refinement, plasticity, layer 4

## Abstract

Sensory cortices do not work in isolation. The functional responses of neurons in primary sensory cortices can be affected by activity from other modalities. For example, short-term visual deprivations, or dark exposure (DE), leads to enhanced neuronal responses and frequency selectivity to sounds in layer 4 (L4) of primary auditory cortex (A1). Circuit changes within A1 likely underlie these changes. Prior studies revealed that DE enhanced thalamocortical transmission to L4 in A1. Because the frequency selectivity of L4 neurons is determined by both thalamocortical and intracortical inputs, changes in intralaminar circuits to L4 neurons might also contribute to improved sound responses. We thus investigated in mouse A1 whether intracortical circuits to L4 cells changed after DE. Using in vitro whole-cell patch recordings in thalamocortical slices from mouse auditory cortex, we show that DE can lead to refinement of interlaminar excitatory as well as inhibitory connections from L2/3 to L4 cells, manifested as a weakening of these connections. The circuit refinement is present along the tonotopic axis, indicating reduced integration along the tonotopic axis. Thus, cross-modal influences may alter the spectral and temporal processing of sensory stimuli in multiple cortical layers by refinement of thalamocortical and intracortical circuits.

## Visual Abstract

**Figure:**
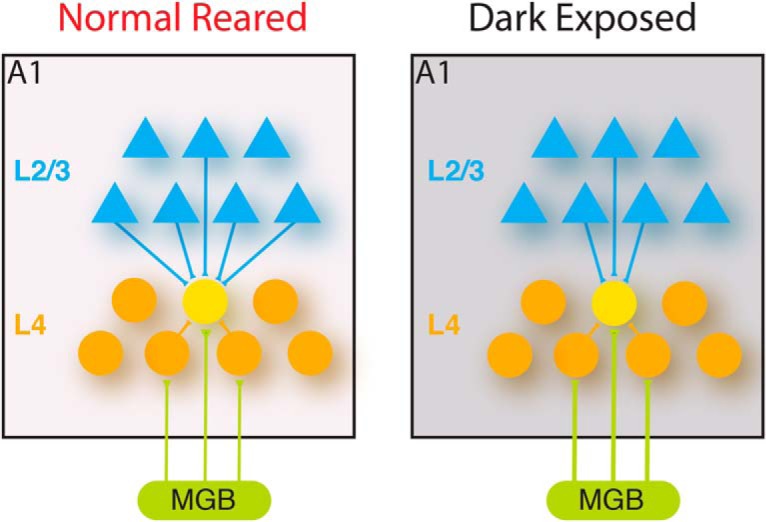

## Significance Statement

Temporary visual deprivation leads to sharper frequency selectivity and increased sensitivity of thalamorecipient neurons in layer 4 (L4) of primary auditory cortex (A1). Although thalamocortical synapses in A1 are strengthened after visual deprivation, the intracortical circuit changes underlying the functional changes in L4 are poorly understood. We here investigated the functional microcircuits targeting L4 neurons. We show that visual deprivations cause a spatial refinement of interlaminar excitatory and inhibitory connections from L2/3 to L4 cells but not within L4. The circuit refinement is present along the tonotopic axis, indicating reduced integration along the tonotopic axis. Our findings show that cross-modal influences can impact the processing of sensory stimuli in L4 by adjusting both thalamocortical and intracortical circuits.

## Introduction

Our perception of the world relies on the integration of inputs from multiple senses, with sensory inputs from different modalities being integrated at different stages of processing. The interaction of the different modalities can be uncovered during the loss of a sensory modality, which often leads to enhanced function of one or more of the remaining senses in a process often termed “cross-modal plasticity” (Bavelier and Neville, 2002; Lee and Whitt, 2015). The best-studied group of individuals are the early or late blind, who can show enhanced performance in the remaining senses, for example better sound localization (Lessard et al., 1998; Röder et al., 1999) and pitch discrimination (Gougoux et al., 2004), than sighted individuals. These behavioral results suggest that the absence of vision may trigger changes in circuits underlying auditory perception. There is accumulating evidence that even primary sensory cortices receive information from other sensory systems. These inputs mainly activate the superficial layers of a primary sensory cortex (Lakatos et al., 2007; Iurilli et al., 2012; Ibrahim et al., 2016), are thought to be important for multisensory integration under normal conditions (Schroeder and Foxe, 2005; Ghazanfar and Schroeder, 2006), and have the ability to trigger profound circuit plasticity. Because thalamorecipient layer 4 (L4) cells receive input from the superficial layers (Barbour and Callaway, 2008; Kratz and Manis, 2015), multisensory inputs might thus sculpt circuits in thalamorecipient layers. Indeed, after the critical period, depriving mice of vision by dark exposure (DE) for ∼ 1 week alters the sound-evoked responses in layer 4 (L4) of primary auditory cortex (A1; Petrus et al., 2014). L4 cells responded more robustly to sounds, consistent with increased thalamocortical transmission after DE (Petrus et al., 2014). L4 neurons also showed increased frequency selectivity (Petrus et al., 2014). Because frequency selectivity tuning of A1 neurons depends on intracortical circuits (Li et al., 2013, 2014), increased selectivity suggests that intracortical circuits to L4 neurons were altered after DE. Because *in vitro* studies showed that a period of DE can refine ascending and intralaminar excitatory and inhibitory circuits to L2/3 neurons (Meng et al., 2015), we speculated that DE could also alter intracortical circuits to L4 neurons and that such circuit changes could contribute to the increased frequency selectivity.

L4 cells in A1 receive inputs from within L4, and these inputs can be patchy (Barbour and Callaway, 2008; Zhao et al., 2009; Kratz and Manis, 2015), similar to intralaminar inputs to L2/3 cells (Watkins et al., 2014). Additional inputs to L4 cells originate in L2/3 as well as weak projection from L5/6 (Barbour and Callaway, 2008; Zhao et al., 2009). In particular, because *in vitro* recordings from L4 had shown that intralaminar connections to L4 neurons are strengthened (Petrus et al., 2015), we speculated that interlaminar connections to L4 neurons might change in the opposite manner.

To identify which microcircuits in L4 A1 neurons are affected by visual experience, we use laser-scanning photostimulation (LSPS) to map spatially the connectivity of excitatory and inhibitory inputs to L4 neurons to determine whether visual deprivation alters their circuit topology. We find that 6–8 d of dark rearing alters the spatial pattern of both excitatory and inhibitory interlaminar connections originating in L2/3. Excitatory and inhibitory inputs originating from L2/3 were confined to a smaller area along the rostro-caudal tonotopic axis, indicating refinement of lateral connections consistent with increased spectral selectivity. Moreover, inputs from L2/3 were weaker, indicating an increase in feed-forward processing of L4. Together, our results show that DE can refine the intracortical circuits in multiple layers of A1 to facilitate enhanced spectro-temporal processing of sound stimuli.

## Methods

### Animals

All procedures followed the University of Maryland College Park animal use regulations. Male and female C57BL/6J mice (Jackson Laboratory) were raised in 12-h light/12-h dark conditions. At postnatal day 21 (P21)–P22, mice (two to three mice from established litters and single gender per cage) were dark exposed (DE) for 6–8 d. Age-matched controls remained in normal light conditions (NR).

### Slice preparation

Mice are deeply anesthetized with isoflurane (Halocarbon). A block of brain containing A1 and the medial geniculate nucleus (MGN) is removed, and thalamocortical slices (500 μm thick) are cut on a vibrating microtome (Leica) in ice-cold ACSF containing (in mm) 130 NaCl, 3 KCl, 1.25 KH_2_PO_4_, 20 NaHCO_3_, 10 glucose, 1.3 MgSO_4_, and 2.5 CaCl_2_ (pH 7.35–7.4, in 95% O_2_/5% CO_2_). For A1 slices, the cutting angle is ∼15 degrees from the horizontal plane (lateral raised; Cruikshank et al., 2002; Zhao et al., 2009; Meng et al., 2015). Slices are incubated for 1 h in ACSF at 30°C and then kept at room temperature. For recording, slices are held in a chamber on a fixed-stage microscope (Olympus BX51) and superfused (2–4 ml/min) with high-Mg ACSF recording solution at room temperature to reduce spontaneous activity in the slice. The recording solution contained (in mm) 124 NaCl, 5 KCl, 1.23 NaH_2_PO_4_, 26 NaHCO_3_, 10 glucose, 4 MgCl_2_, and 4 CaCl_2_. The location of the recording site in A1 was identified by landmarks (Cruikshank et al., 2002; Zhao et al., 2009; Meng et al., 2015).

### Electrophysiology

**Figure 1.**
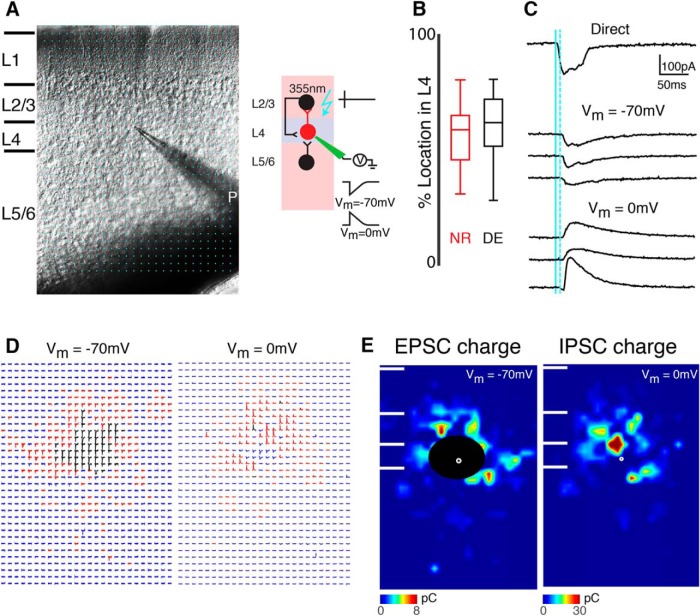
LSPS to map intracortical connections to L4 cells. ***A***, Left, infrared image of brain slice with patch pipette on L4 neuron. Stimulation grid is indicated by blue dots. Right, schematic of LSPS experiment. Whole-cell patch-clamp recordings are made from L4 neurons. Cells are held at –70 and 0 mV. Laser pulses (355 nm) are targeted to an array of locations in the slice. Traces on right, activated cells fire action potentials (top), and if a connection exists to the patched L4 neuron, evoked EPSCs and IPSCs are recorded (bottom). ***B***, The relative position of patched cells within L4. 0 refers to the border with L5 and 100 refers to the border with L3. Cells were sampled from the middle of layer 4 in NR and DE animals (*p* = 3.05 × 10^–1^). ***C***, Whole-cell voltage clamp recordings at holding potentials of −70 mV (top) or 0 mV (bottom) distinguish between photostimulation-evoked excitatory and inhibitory currents. Shown are traces obtained with photostimulation at different locations. Solid blue line indicates time of photostimulation; dashed blue line marks 8-ms poststimulus, which is the minimal latency for synaptic responses. ***D***, Traces obtained by LSPS when holding one L4 neuron at −70 and 0 mV, respectively. Traces showing large-amplitude direct responses are shown in black. The responses that have latencies between 8 and 50 ms are shown in red. Otherwise, the traces are shown in blue. ***E***, Pseudocolor maps show PSC charge at each stimulus location for the example cell in ***D***. Direct responses indicated were set to zero (overlaid by black area). White filled circle marks the soma location. Horizontal bars indicate layer borders.

Whole-cell recordings are performed with a patch clamp amplifier (Multiclamp 700B, Molecular Devices) using pipettes with input resistance of 4–9 MΩ. Cells targeted for recording are located in an area of A1 overlying the rostral flexure of the hippocampus. Data acquisition is performed by National Instruments AD boards and custom software (Ephus; Suter et al., 2010), which is written in Matlab (Mathworks) and adapted to our setup. Voltages are corrected for an estimated junction potential of 10 mV. Electrodes are filled with (in mm) 115 cesium methanesulfonate (CsCH_3_SO_3_), 5 NaF, 10 EGTA, 10 HEPES, 15 CsCl, 3.5 MgATP, and 3 QX-314 (pH 7.25, 300 mOsm). Biocytin or neurobiotin (0.5%) is added to the electrode solution as needed. Series resistances were typically 20–25 MΩ. For photostimulation, 0.5–1 mm caged glutamate [*N*-(6-nitro-7-coumarinylmethyl)-l-glutamate; Ncm-Glu; Kao, 2006; Muralidharan et al., 2016] is added to the ACSF. Without UV light, this compound has no effect on neuronal activity (Kao, 2006; Muralidharan et al., 2016). UV laser light (500 mW, 355 nm, 1-ms pulses, 100-kHz repetition rate, DPSS) is split by a 33% beam splitter (CVI Melles Griot), attenuated by a Pockels cell (Conoptics), gated with a laser shutter (NM Laser), and coupled into a microscope via scan mirrors (Cambridge Technology) and a dichroic mirror. The laser beam in LSPS enters the slice axially through the objective (Olympus 10×, 0.3 NA/water) and has a diameter of <20 μm. Laser power at the sample is < 25 mW. We typically stimulate up to 40 × 35 sites spaced 30 μm apart, enabling us to probe areas of 1 mm^2^; such dense sampling reduces the influence of potential spontaneous events. Repeated stimulation yielded essentially identical maps. Stimuli are applied at 0.5–1 Hz. Analysis was performed essentially as described previously with custom software written in Matlab (Meng et al., 2014, 2015). Activation profiles of neurons were produced by recording in cell-attached mode while mapping the same region and recording action potentials. To detect monosynaptically evoked postsynaptic currents (PSCs), we detected PSCs with onsets in an ∼50-ms window after the stimulation (Fig. 1*C*). This window was chosen based on the observed spiking latency under our recording conditions (Meng et al., 2015). Our recordings are performed at room temperature and in high-Mg^2+^ solution to reduce the probability of polysynaptic inputs. We measured both peak amplitude and transferred charge; transferred charge was measured by integrating the PSC. Although the transferred charge might include contributions from multiple events, our prior studies showed a strong correlation between these measures (Viswanathan et al., 2012; Meng et al., 2014, 2015). Traces containing a short-latency (<8 ms) “direct” response were discarded from the analysis (Fig. 1*E*, black patches in color-coded maps), as were traces that contained longer-latency inward currents of long duration (>50 ms). These currents could sometimes be seen in locations surrounding (<100 μm) areas that gave a direct response. Occasionally, some of the direct responses contained synaptic evoked responses that we did not separate out, leading to an underestimation of local short-range connections. Cells that did not show any large (>100 pA) direct responses were excluded from the analysis, as these could be astrocytes. It is likely that the observed PSCs at each stimulus location represent the activity of multiple presynaptic cells. Layer boundaries were determined from the infrared pictures.

### Statistics

Results are plotted as means ± SD unless otherwise indicated. Populations are compared with a rank sum or Student’s *t* test (based on Lilliefors test for normality), and the PSTH variance comparison is done with *F* test and deemed significant if *p* < 0.05.

## Results

We use laser-scanning photostimulation (LSPS) with caged glutamate (Shepherd et al., 2003; Meng et al., 2014, 2015) to map spatially the connectivity of excitatory and inhibitory inputs to A1 neurons to determine whether temporary visual deprivation alters circuits in A1 (Fig. 1*A*). We thus compare mice raised in normal light conditions (NR) with mice that were dark exposed (DE) from 1 wk starting at ∼P21 and mapped cells from NR and DE animals at P28–P30. Cells from NR and DE were located at similar laminar positions (Fig. 1*B*; *p* = 0.3). We previously showed by cell-attached recordings that DE does not cause increased excitability or increased sensitivity of L4 and L2/3 cells to glutamate (e.g., by redistribution of GluRs to the soma or proximal dendrites; Meng et al., 2015).

### Interlaminar excitatory connections to A1 L4 neurons change after DE

We first investigated whether the spatial pattern of intra- and interlaminar connectivity to L4 neurons is altered after DE. To visualize the spatial pattern of excitatory inputs of each cell, we performed whole-cell patch recordings and targeted the laser pulse to multiple distinct stimulus locations and record the resulting membrane currents (Fig. 1*A*). If the neuron activated by the laser pulse was connected to the recorded neuron, then evoked PSCs were observed. By holding cells at a membrane potential of –70 mV (∼*E*_Cl_) we can isolate EPSCs (Fig. 1*A*,*C*). We then targeted the laser pulse to multiple distinct stimulus locations and recorded the resulting membrane currents. The targeted stimulus locations spanned the entire extent of A1, thus enabling us to probe the entire 2D connection pattern of excitatory inputs to a given cell over ∼1 mm^2^ (Fig. 1*A*). Because activation of the cell body and proximal dendrites causes a large-amplitude short-latency direct event and synaptic currents have a distinct latency (>8 ms), we can separate them by latency criteria (Fig. 1*C*).

**Figure 2.**
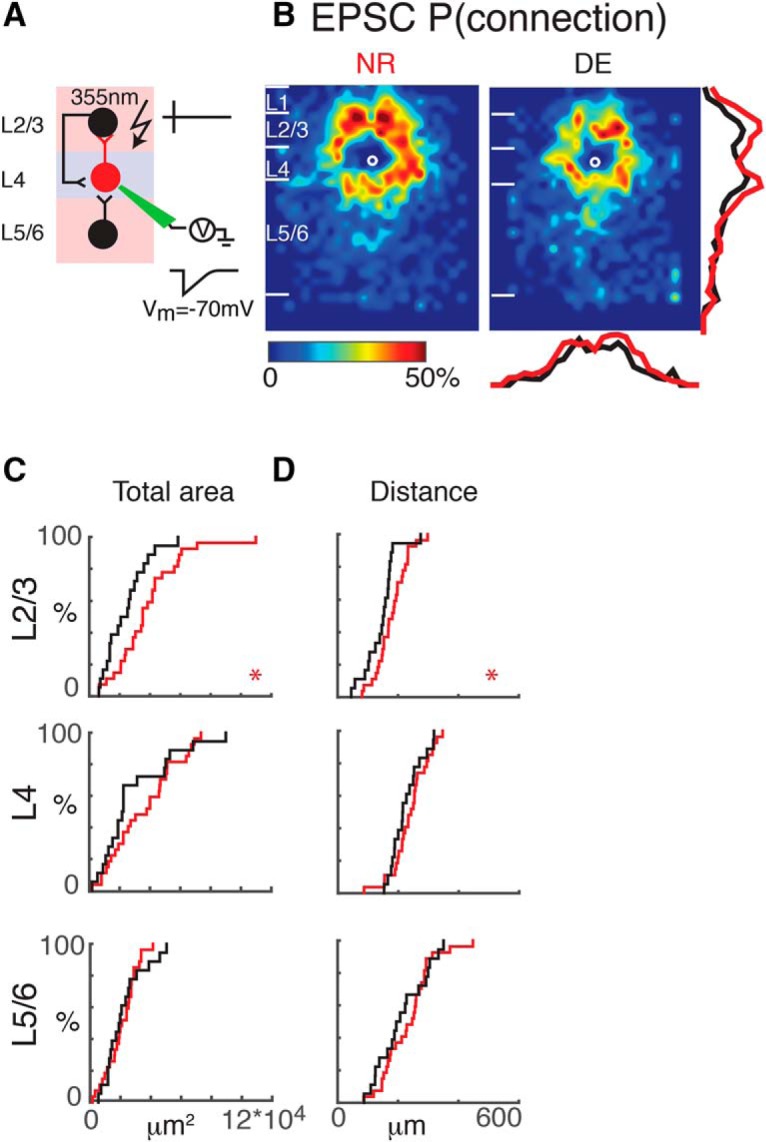
Interlaminar cortical excitatory connections to L4 cells refine with DE. ***A***, Schematic of LSPS experiment. Whole-cell patch-clamp recordings are made from L4 neurons. Cells are held at –70 mV. Laser pulses (355 nm) are targeted to an array of locations in the slice. Traces on right, activated cells fire action potentials (top), and if a connection exists to the patched L4 neuron, evoked EPSCs are recorded (bottom). ***B***, Average maps (aligned to soma, white circle) of connection probability for excitatory connections in NR (left) and DE (right) animals. Connection probability is encoded according to the pseudocolor scale. White horizontal lines indicate averaged laminar borders and are 100 μm long. Traces at the right of the DE panel the laminar marginal distributions (red for NR and black for DE). Traces at the bottom of the DE panel are the columnar marginal distributions. Note that NR and DE maps and distributions appear different. ***C***, Distributions of area of input originating from L2/3 (top), L4 (middle), and L5/6 (bottom) of NR (red) or DE (black) animals. *, *p* < 0.05. The *p* values for the total area from L2/3, L4, and L5/6 are 0.02 (NR: mean = 3.8 × 10^4^ μm^2^, std = 2.2 × 10^4^ μm^2^; DE: mean = 2.4 × 10^4^ μm^2^, std = 1.4 × 10^4^ μm^2^), 0.74 (NR: mean = 2.1 × 10^4^ μm^2^, std = 1.0 × 10^4^ μm^2^; DE: mean = 2.2 × 10^4^ μm^2^, std = 1.3 × 10^4^ μm^2^), and 0.32 (NR: mean = 3.6 × 10^4^ μm^2^, std = 2.1 × 10^4^ μm^2^; DE: mean = 2.9 × 10^4^ μm^2^, std = 2.4 × 10^4^ μm^2^), respectively. ***D***, Distributions of the distance of 80% of input to each L4 cell originating from L2/3 (top), L4 (middle), and L5/6 (bottom) of NR (red) or DE (black) animals. We calculated the laminar radius that covers 80% of inputs inside each layer and plotted the CDFs of the radius. *, *p* < 0.05. All comparisons were done with Wilcoxon rank-sum test or Student’s *t* test. The *p* values for the average 80% distance from L2/3, L4, and L5/6 are 0.028 (NR: mean = 179.2 μm, std = 51 μm; DE: mean = 143.6 μm, std = 52.3 μm), 0.40 (NR: mean = 239.4 μm, std = 58.7 μm; DE: mean = 225.2 μm, std = 49.9 μm), and 0.39 (NR: mean = 233.5 μm, std = 79.8 μm; DE: mean = 212.2 μm, std = 81.0 μm), respectively.

We mapped L4 cells (*n* = 46 cells) in A1 and examined the connection pattern of excitatory inputs. L4 cells in normal reared animals (NR, *n* = 27 cells) received excitatory input from within L4 as well as from L2/3 and L5/6 (Fig. 1*D*, *E*), consistent with prior studies (Barbour and Callaway, 2008; Zhao et al., 2009; Kratz and Manis, 2015). To analyze connectivity pattern changes over the population of cells, individual LSPS maps were aligned to the cell body position and averaged; the result is a spatial map of connection probability (Fig. 2*A*,*B*). These maps showed that L4 cells were connected to other L4 cells up to 500 μm apart. Because our thalamocortical slices contain the tonotopic axis, this indicates that L4 cells can integrate inputs that are more than one octave above or below the cell’s best frequency (BF).

Altered synaptic connectivity can be manifested as altered occurrence of connections as well as changes in the strength of existing connections. We therefore analyzed the spatial connection probability and the spatial connection strength separately. When qualitatively comparing NR to DE, we find that after DE there are distinct differences in excitatory inputs to L4 neurons (Fig. 2*B*, NR: *n* = 27; DE: *n* = 19). Overall there seems to be a reduction in connection probability for inputs originating from L2/3 and L4. Although average connection maps allow a coarse assessment of changes, detailed changes in connection profiles cannot be extracted when the individual connection profiles are diverse (Meng et al., 2015). Therefore, we analyzed properties of the connection patterns for each individual cell in detail and compared these properties over the population. To quantify the laminar changes, we identified laminar borders for each cell from the differential interference contrast (DIC) images and calculated the input profile from each layer. To visualize and quantify the differences between cells, we determined the total area in each layer where stimulation evoked EPSCs in L4 neurons. We found that after DE, the area of excitatory inputs originating from L2/3 but not L4 was decreased, suggesting a pruning/refinement of functional interlaminar but not intralaminar connections (Fig. 2*C*). To further analyze the functional connectivity, we calculated the laminar distance from each functionally connected stimulation site to the recorded cells. After DE, inputs from L2/3 originated from closer distances than in NR (Fig. 2*D*), consistent with pruning or refinement of interlaminar inputs.

### Interlaminar inhibitory connections to A1 L4 neurons change after DE

**Figure 3.**
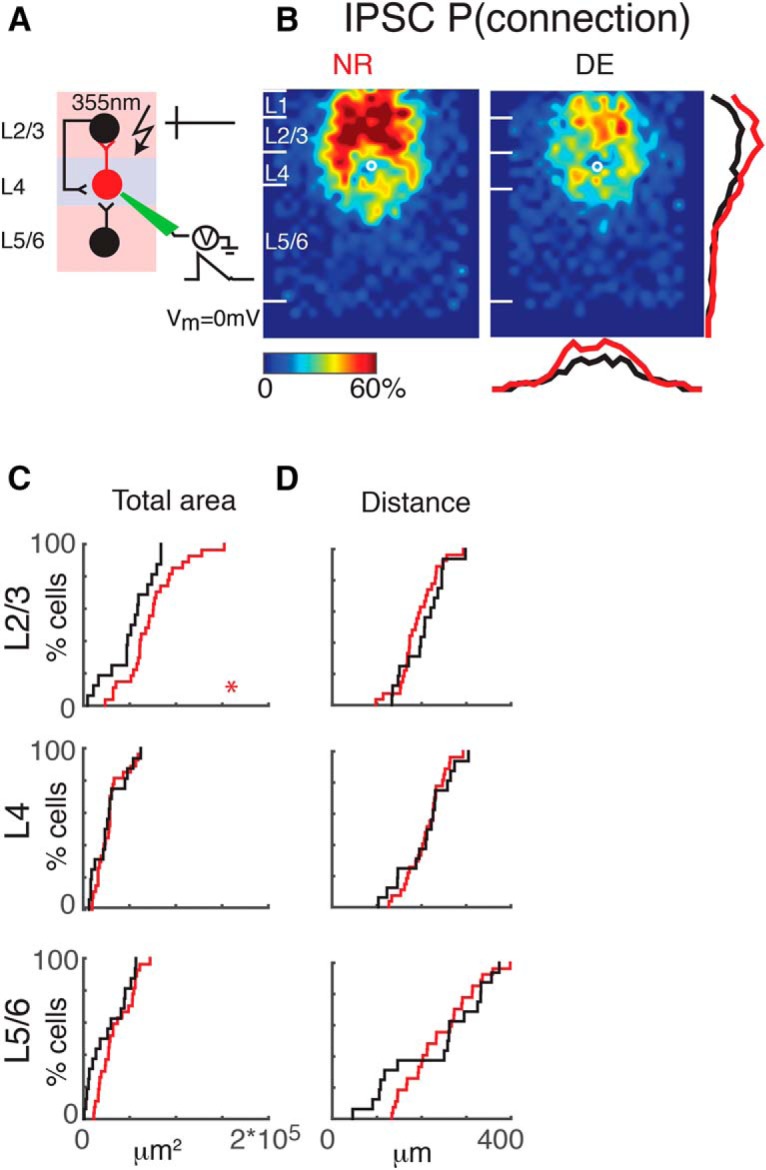
Interlaminar cortical inhibitory connections to L4 cells refine. ***A***, Schematic of LSPS experiment. Whole-cell patch-clamp recordings are made from L4 neurons. Cells are held at 0 mV. Laser pulses (355 nm) are targeted to an array of locations in the slice. Traces on right, activated cells fire action potentials (top), and if a connection exists to the patched L4 neuron, evoked IPSCs are recorded (bottom). ***B***, Average maps (aligned to soma, white circle) of connection probability for inhibitory connections in NR (left) and DE (right) animals. Connection probability is encoded according to the pseudocolor scale. White horizontal lines indicate averaged laminar borders and are 100 μm long. Traces at the right of the DE panel are the laminar marginal distributions (red for NR and black for DE). Traces at the bottom of the DE panel are the columnar marginal distributions. Note that NR and DE maps and distributions appear different. ***C***, Distributions of area of input originating from L2/3 (top), L4 (middle), and L5/6 (bottom) of NR (red) or DE (black) animals. *, *p* < 0.05. The *p* values for the total area from L2/3, L4, and L5/6 are 0.02 (NR: mean = 7.3 × 10^4^ μm^2^, std = 2.9 × 10^4^ μm^2^; DE: mean = 5.1 × 10^4^ μm^2^, std = 2.5 × 10^4^ μm^2^), 0.82 (NR: mean = 2.8 × 10^4^ μm^2^, std = 1.5 × 10^4^ μm^2^; DE: mean = 2.7 × 10^4^ μm^2^, std = 1.7 × 10^4^ μm^2^), and 0.14 (NR: mean = 3.5 × 10^4^ μm^2^, std = 1.8 × 10^4^ μm^2^; DE: mean = 2.6 × 10^4^ μm^2^, std = 2.41 × 10^4^ μm^2^), respectively. ***D***, Distributions of the distance of 80% of input to each L4 cell originating from L2/3 (top), L4 (middle), and L5/6 (bottom) of NR (red) or DE (black) animals. *, *p* < 0.05. All comparisons were done with Wilcoxon rank-sum test or Student’s *t* test. The *p* values for the average 80% distance from L2/3, L4, and L5/6 are 0.34 (NR: mean = 190.4 μm, std = 42.7 μm; DE: mean = 204.0 μm, std = 56.2 μm), 0.95 (NR: mean = 209.3 μm, std = 41.0 μm; DE: mean = 208.4 μm, std = 56.2 μm), and 0.74 (NR: mean = 237.9 μm, std = 74.7 μm; DE: mean = 228.6 μm, std = 108.8 μm), respectively.

Our results show a remodeling of excitatory connections. We next investigated whether inhibitory connections also change after DE. We mapped inhibitory connections by holding cells at 0 mV (∼*E*_glut_; Figs. 1*D*, *E*, and 3*A*). Average maps of connection probability and connection strength appeared different after DE, in that the cortical area giving rise to inhibitory responses decreased (Fig. 3*B*). This was confirmed quantitatively: the total area generating inhibitory input in L2/3 was reduced after DE compared with NR controls (Fig. 3*C*). However, in contrast to the excitatory inputs, analysis of the distance from where inputs could be evoked did not show differences after DE (Fig. 3*D*). This indicates that the refinement of inhibitory inputs is due to refinement within L2/3 with inhibitory inputs originating from a smaller sublamina within L2/3 after DE. Taken together, the above results demonstrate refinement of both excitatory and inhibitory connections originating in L2/3 after DE while inputs from L4 and L5/6 did not change.

### The strength of interlaminar connections to A1 L4 neurons changes after DE

**Figure 4.**
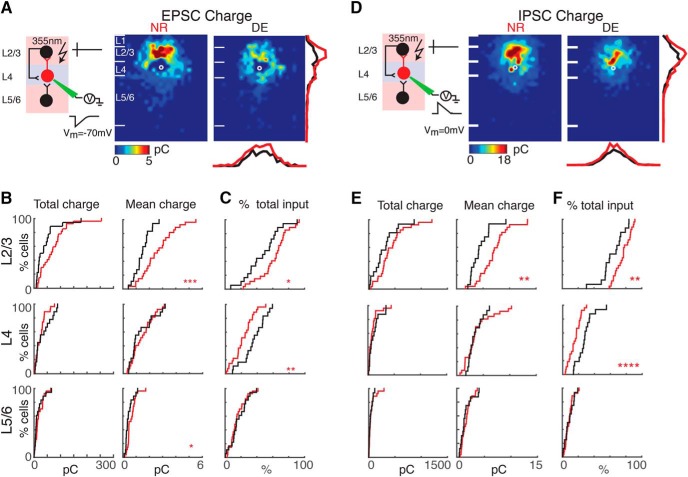
The EPSC and IPSC charge of interlaminar cortical connections to L4 cells decreases. ***A***, Average maps (aligned to soma, white circle) of connection strength (transferred charge) for excitatory inputs in NR (left) and DE (right) animals. Averages are calculated only for stimulation sites that evoked responses in >10% of cells in our sample. Connection strength is encoded according to the pseudocolor scale. White horizontal lines indicate averaged laminar borders and are 100 μm long. Traces at the right of DE panel are the laminar marginal distributions (red for NR and black for DE). Traces at the bottom of the DE panel are the columnar marginal distributions. Note that NR and DE maps and distributions appear different. ***B***, Distributions of total (left) and mean EPSC (right) input charge originating from L2/3 (top), L4 (middle), and L5/6 (bottom) of NR (red) or DE (black) animals. *, *p* < 0.05; ***, *p* < 0.01. The *p* values for the total charge from L2/3, L4, and L5/6 are 0.06 (NR: mean = 71 pC, std = 52.6 pC; DE: mean = 42.6 pC, std = 42.4 pC), 0.17 (NR: mean = 25 pC, std = 19.5 pC; DE: mean = 35.2 pC, std = 29.7 pC), and 0.37 (NR: mean = 18.5 pC, std = 15.6 pC; DE: mean = 15.8 pC, std = 17.3 pC), respectively. The *p* values for the mean EPSC charge from L23, L4, and L5/6 are 8.1 × 10^−4^ (NR: mean = 2.59 pC, std = 1.23 pC; DE: mean = 1.46 pC, std = 0.6 pC), 0.73 (NR: mean = 1.45 pC, std = 0.8 pC; DE: mean = 1.36 pC, std = 0.91 pC), and 0.02 (NR: mean = 0.63 pC, std = 0.33 pC; DE: mean = 0.45 pC, std = 0.28 pC), respectively. All comparisons were done with Wilcoxon rank-sum test or Student’s *t* test. ***C***, Distributions of fractional EPSC charge originating from L2/3 (top), L4 (middle), and L5/6 (bottom) for cells from NR (red) or DE (black) animals. L2/3: *p* = 0.015 (NR: mean = 0.62, std = 0.18; DE: mean = 0.47, std = 0.21), L4: *p* = 0.002 (NR: mean = 0.22, std = 0.13; DE: mean = 0.37, std = 0.15), and L5/6: *p* = 0.67 (NR: mean = 0.16, std = 0.1; DE: mean = 0.17, std = 0.11). ***D***, Average maps (aligned to soma, white circle) of connection strength (transferred charge) for inhibitory inputs in NR (left) and DE (right) animals. Averages are calculated only for stimulation sites that evoked responses in >10% of cells in our sample. Note that NR and DE maps and distributions appear different. ***E***, Distributions of total (left) and mean (right) IPSC input charge originating from L2/3 (top), L4 (middle), and L5/6 (bottom) of NR (red) or DE (black) animals. *, *p* < 0.05; ***, *p* < 0.01. The *p* values of the total IPSC charge from L2/3, L4, and L5/6 are 0.11 (NR: mean = 413.8 pC, std = 265.7 pC; DE: mean = 285.2 pC, std = 227.7 pC), 0.37 (NR: mean = 87.4 pC, std = 94.3 pC; DE: mean = 115.3 pC, std = 104 pC), and 0.41 (NR: mean = 51.4 pC, std = 63.7 pC; DE: mean = 37.2 pC, std = 35.1 pC), respectively. The *p* values of the mean IPSC charge from L23, L4, and L5/6 are 1.2 × 10^−3^ (NR: mean = 7.01 pC, std = 2.69 pC; DE: mean = 4.35 pC, std = 1.87 pC), 0.67 (NR: mean = 3.71 pC, std = 2.53 pC; DE: mean = 3.42 pC, std = 1.33 pC), and 0.67 (NR: mean = 1.58 pC, std = 0.88 pC; DE: mean = 1.44 pC, std = 01.13 pC), respectively. All comparisons were done with Wilcoxon rank-sum test or Student’s *t* test. ***F***, Distributions of fractional IPSC charge originating from L2/3 (top), L4 (middle), and L5/6 (bottom) for cells from NR (red) or DE (black) animals: *p* = 0.001 (NR: mean = 0.76, std = 0.1; DE: mean = 0.64, std = 0.14), L4: *p* = 4.12*10^−5^ (NR: mean = 0.15, std = 0.07; DE: mean = 0.28, std = 0.11), and L5/6: *p* = 0.961 (NR: mean = 0.08, std = 0.05; DE: mean = 0.09, std = 0.06).

**Figure 5.**
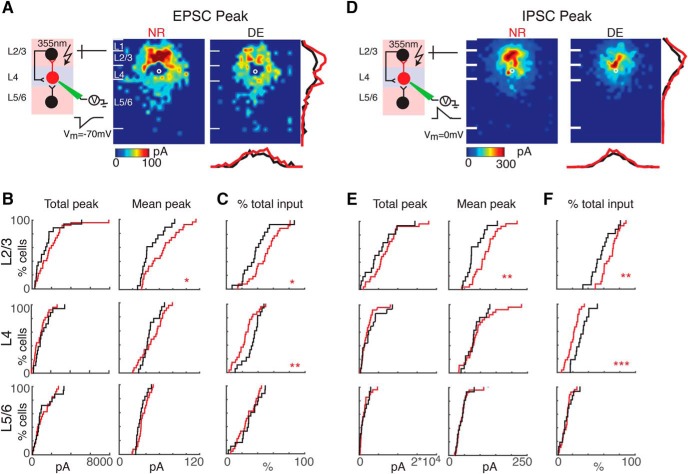
The EPSC and IPSC peak amplitude of interlaminar cortical connections to L4 cells decreases. ***A***, Average maps (aligned to soma, white circle) of peak amplitude for excitatory inputs in NR (left) and DE (right) animals. Averages are calculated only for stimulation sites that evoked responses in >10% of cells in our sample. Peak amplitude is encoded according to the pseudocolor scale. White horizontal lines indicate averaged laminar borders and are 100 μm long. Traces at the right of the DE panel are the laminar marginal distributions (red for NR and black for DE). Traces at the bottom of the DE panel are the columnar marginal distributions. Note that NR and DE maps and distributions appear different. ***B***, Distributions of total (left) and mean (right) EPSC peak amplitude originating from L2/3 (top), L4 (middle), and L5/6 (bottom) of NR (red) or DE (black) animals. *, *p* < 0.05; **, *p* < 0.01. The *p* values for the total peak from L2/3, L4, and L5/6 are 0.25 (NR: mean = 1.89 ×10^3^ pA, std = 1.55 × 10^3^ pA; DE: mean = 1.38 × 10^3^ pA, std = 1.22 × 10^3^ pA), 0.24 (NR: mean = 9.05 × 10^2^ pA, std = 6.81 × 10^2^ pA; DE: mean = 1.17 × 10^3^ pA, std = 8.23 × 10^2^ pA), and 0.99 (NR: mean = 1.11 × 10^3^ pA, std = 8.39 × 10^2^ pA; DE: mean = 1.11 × 10^3^ pA, std = 9.98 × 10^2^ pA), respectively. The *p* values for the mean peak from L2/3, L4, and L5/6 are 0.02 (NR: mean = 63.4 pA, std = 24.3 pA; DE: mean = 47.4 pA, std = 16.9 pA), 0.42 (NR: mean = 49.4 pA, std = 16.8 pA; DE: mean = 45.7 pA, std = 11.1 pA), and 0.2 (NR: mean = 35.4 pA, std = 8.15 pA; DE: mean = 32.3 pA, std = 6.85 pA), respectively. All comparisons were done with Wilcoxon rank-sum test or Student’s *t* test. ***C***, Distributions of fractional EPSC amplitude originating from L2/3 (top), L4 (middle), and L5/6 (bottom) for cells from NR (red) or DE (black) animals. L2/3: *p* = 0.03 (NR: mean = 0.50, std = 0.18; DE: mean = 0.37, std = 0.18), L4: *p* = 0.009 (NR: mean = 0.23, std = 0.12; DE: mean = 0.33, std = 0.10), and L5/6: *p* = 0.5(NR: mean = 0.26, std = 0.12; DE: mean = 0.29, std = 0.14). ***D***, Average maps (aligned to soma, white circle) of connection strength (transferred peak) for inhibitory inputs in NR (left) and DE (right) animals. Averages are calculated only for stimulation sites that evoked responses in >10% of cells in our sample. Traces at the right of the DE panel are the laminar marginal distributions (red for NR and black for DE). Traces at the bottom of the DE panel are the columnar marginal distributions. Note that NR and DE maps and distributions appear different. ***E***, Distributions of total (left) and mean (right) IPSC peak amplitude originating from L2/3 (top), L4 (middle), and L5/6 (bottom) of NR (red) or DE (black) animals. *, *p* < 0.05; ***, *p* < 0.01. The *p* values for the total peak from L23, L4, and L5/6 are 0.22 (NR: mean = 6.74 ×10^3^ pA, std = 3.83 × 10^3^ pA; DE: mean = 5.24 × 10^3^ pA, std = 3.85 × 10^3^ pA), 0.28 (NR: mean = 2.01 × 10^3^ pA, std = 1.63 × 10^3^ pA; DE: mean = 2.69 × 10^3^ pA, std = 2.40 × 10^3^ pA), and 0.91 (NR: mean = 1.23 × 10^3^ pA, std = 1.10 × 10^3^ pA; DE: mean = 1.19 × 10^3^ pA, std = 1.02 × 10^3^ pA), respectively. The *p* values for the mean peak from L23, L4, and L5/6 are 0.004 (NR: mean = 118 pA, std = 38.4 pA; DE: mean = 82.8 pA, std = 30.9 pA), 0.42 (NR: mean = 90.1 pA, std = 46.0 pA; DE: mean = 79.8 pA, std = 27.3 pA), and 0.86 (NR: mean = 41.5 pA, std = 18.3 pA; DE: mean = 40.5 pA, std = 15.5 pA), respectively. All comparisons were done with Wilcoxon rank-sum test or Student’s *t* test. ***F***, Distributions of fractional IPSC amplitude originating from L2/3 (top), L4 (middle), and L5/6 (bottom) for cells from NR (red) or DE (black) animals. L2/3: *p* = 4.7 × 10^−3^ (NR: mean = 0.68, std = 0.10; DE: mean = 0.57, std = 0.13), L4: *p* = 2.95 × 10^−4^ (NR: mean = 0.20, std = 0.07; DE: mean = 0.30, std = 0.09), and L5/6: *p* = 0.83 (NR: mean = 0.12, std = 0.06; DE: mean = 0.12, std = 0.07). All comparisons were done with Wilcoxon rank-sum test or Student’s *t* test.

Circuit changes can involve changes in connection probability as well as changes in synaptic strength. Therefore, we next investigated whether the strength of events evoked from each layer changed after DE. Because synaptic events can change in amplitude as well as duration, we calculated both charge and peak amplitude of the evoked EPSCs. We found that the mean EPSC charge, as well as EPSC amplitude of events originating from L2/3 and L5/6, decreased after DE (Figs. 4*A*, *B* and 5*A*, *B*). Because the amplitude of events originating in L4 did not change, the fractional charge L4 cells received from within L4 as opposed to interlaminar inputs increased (Figs. 4*C* and 5*C*). The laminar changes in IPSC strength after DE mirrored the changes in EPSC strength. The average charge and amplitude of uncaging evoked IPSC was decreased in L2/3 after DE (Figs. 4*D*, *E* and 5*D*, *E*), leading to a relative increase in input from L4 (Figs. 4*F* and 5*F*). Together, these results demonstrate a weakening of interlaminar excitatory and inhibitory inputs from L2/3 to L4 neurons after DE.

### The balance of excitation and inhibition from L2/3 to L4 changes after DE

**Figure 6.**
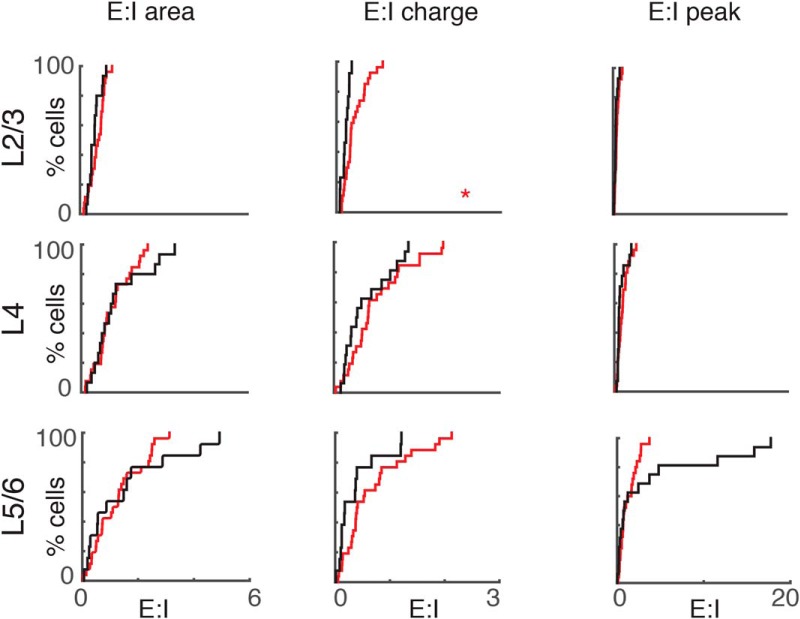
The balance of excitation and inhibition of L2/3 inputs to L4 is reduced. Cumulative distributions (CDFs) of excitation/inhibition (EI) area ratio (left), charge ratio (middle), and peak amplitude ratio (right) from L2/3 (top), L4 (middle), and L5/6 (bottom) in NR and DE cells. The charge ratio of L2/3 inputs decreased after DE (EI area ratio: L2/3: *p* = 0.2, L4: *p* = 0.53, L5/6: *p* = 0.55; EI charge ratio: L2/3: *p* = 0.007, L4: *p* = 0.26, L5/6: *p* = 0.08; EI amplitude ratio: L2/3: *p* = 0.12, L4: *p* = 0.29, L5/6: *p* = 0.90). All comparisons were done with Wilcoxon rank-sum test or Student’s *t* test.

DE results in a balanced refinement of excitatory and inhibitory connections to L2/3 neurons (Meng et al., 2015). Because thalamic input to L4 neurons is increased after DE (Petrus et al., 2014), the adjustment of intracortical circuits to L4 neurons might compensate for this additional driving input. We thus investigated whether the changes in the spatial pattern of excitatory and inhibitory connection to L4 neurons occur in a balanced manner. We computed the excitation/inhibition (EI) ratio based on input area, transferred charge, and peak amplitude for every cell. Because we could not assess excitatory input in locations that gave direct responses for excitation, we excluded those stimulus locations in our calculations for both excitation and inhibition. Our calculations showed that the EI ratio for L2/3 inputs decreased after DE (Fig. 6), indicating that L4 neurons received less excitatory input from L2/3. This suggests that increased firing rates in response to sound stimulation after DE are due to increased thalamocortical input (Petrus et al., 2014).

## Discussion

We here show that short-term DE causes a refinement of the functional intracortical circuitry to layer 4 neurons of A1. We found that interlaminar excitatory inputs from L2/3 and L5/6 originate from smaller areas along the rostro-caudal tonotopic axis, indicating a refinement of these connections. DE not only changed excitatory inputs to L4 neurons. Inhibitory connections from L2/3, as well as from L5/6, also originated from reduced areas. Thus, overall, there is a net decrease in the spatial extent of both interlaminar excitation and inhibition to L4 cells. In contrast, intralaminar inputs from within L4 did not change after DE. Because the frequency selectivity of A1 neurons depends on intracortical circuits (Li et al., 2013, 2014), the circuit refinement along the tonotopic axis is consistent with the increasing frequency selectivity of L4 neurons *in vivo* (Petrus et al., 2014). Moreover, our results suggest that interlaminar inputs from L2/3 may help shape the frequency selectivity of L4 neurons. Although we detect refinement of L2/3 to L4 connections, intralaminar connections within L4 were not changed. Neighboring L4 neurons show higher similarity in their frequency selectivity than L2/3 neurons (Bandyopadhyay et al., 2010, Winkowski and Kanold, 2013, and Kanold et al., 2014), suggesting that the refinement we observe in L2/3 decreases connections between neurons of different frequency selectivity. Together with the strengthening of thalamocortical connections to L4 neurons after DE (Petrus et al., 2014), this indicates that DE causes a remodeling of all inputs to L4 neurons in A1 to improve sound processing. Finally, because DE also causes remodeling of A1 circuits in L2/3, our results suggest that A1 processing in general can be highly plastic after the critical period (Meng et al., 2015).

We analyzed A1 circuits using LSPS, which reveals the connections between the photostimulated neurons and the neuron being monitored by patch clamp. Because presynaptic neurons can connect to postsynaptic neurons via multiple individual synapses, the reduction in connection strength from L2/3 to L4 we observed could have been due to fewer synapses between L2/3 and L4 neurons or the weakening of synapses. Analysis of individual synaptic inputs to L4 neurons showed that DE increased synaptic amplitude of intralaminar connections within L4 (Petrus et al., 2015). We did not detect a change in LSPS-evoked amplitude, suggesting that L4 neurons are connected to each other with fewer but stronger synapses. Moreover, the spatial resolution of our LSPS technique is ∼100 µm owing to the direct response. As a result, we cannot measure changes in connections of neurons that are very close to the patched cell, and it is possible that DE strongly affects these very local connections.

As in our prior studies (Goel et al., 2006; He et al., 2012; Petrus et al., 2014; Meng et al., 2015), we here performed our visual deprivation on animals within the critical period for V1 plasticity but after the critical period for A1 plasticity (Barkat et al., 2011; Espinosa and Stryker, 2012). Because we have shown that crossmodal synaptic plasticity occurs in adults (Petrus et al., 2014, 2015), the changes observed here are not likely restricted to the visual deprivation within the V1 critical period.

Our results suggest that the changes after DE include spatial refinement of intracortical inputs to A1 L4 neurons into fewer synapses while thalamocortical synapses strengthen. Thus, the combined effect of these balanced circuit changes will be enhanced transmission of ascending auditory information. The relative increase in feed-forward connectivity by DE may lead to enhancement of spectro-temporal responses in A1 observed in our previous study (Petrus et al., 2014).

Visual deprivation causes pronounced changes in the functional circuits of both L4 and L2/3 in A1. Despite these profound effects, it is unclear how these changes come about. The functional responses in A1 can be modulated by behavior and attention, and these changes are mediated by modulatory and top-down pathways (Kilgard and Merzenich, 1998; Bao et al., 2001; Fritz et al., 2003, 2007; Winkowski et al., 2013, 2017). Because animals in the dark likely pay more attention, an enhanced engagement of these plasticity processes could lead to the observed circuit changes. A nonexclusive further possibility is that the observed changes reflect homeostatic adjustment of latent multisensory processing in A1. Because extrastriate visual cortex can alter A1 activity (Banks et al., 2011), decreased visual activity during DE could lead to a homeostatic rebalancing of auditory circuits. Although our experiments cannot distinguish between these mechanisms, the sum of our observations indicates changes in both excitatory and inhibitory connections consequent to DE in L4 as well as L2/3. Our results thus reveal a powerful effect of cross-modal inputs on the intrinsic circuitry across the different layers of A1.

## Synthesis

Reviewing Editor: Tatiana Pasternak, University of Rochester Decisions are customarily a result of the Reviewing Editor and the peer reviewers coming together and discussing their recommendations until a consensus is reached. When revisions are invited, a fact-based synthesis statement explaining their decision and outlining what is needed to prepare a revision will be listed below. Note: If this manuscript was transferred from JNeurosci and a decision was made to accept the manuscript without peer review, a brief statement to this effect will instead be what is listed below.

The paper is a follow up on a series of well-received studies examining the effects of depriving mice of visual inputs for one week on the intracortical inputs to layer 4 of the primary auditory cortex (A1. It provides further evidence for experience-dependent refinement of intracortical connections by showing that excitatory and inhibitory inputs to layer 4 neurons from other cortical layers are also reduced, although no changes occur in intralaminar layer 4 inputs. The experiments appear to have been carefully conducted using an appropriate methods to interpret the functional changes in A1 neurons that result from dark exposure.
